# Placental and Fetal In Utero Growth Among Fetuses With Congenital Heart Disease

**DOI:** 10.1001/jamanetworkopen.2025.7217

**Published:** 2025-04-24

**Authors:** Marin Jacobwitz, Kushal Kapse, Julius Ngwa, Josepheen De Asis-Cruz, Yao Wu, Mary T. Donofrio, Caitlin McDermott, Adre du Plessis, Catherine Limperopoulos, Nickie Andescavage

**Affiliations:** 1 Developing Brain Institute, Children’s National Hospital, Washington, DC; 2 Division of Cardiology, Children’s National Hospital, Washington, DC; 3 Prenatal Pediatrics Institute, Children’s National Hospital, Washington, DC

## Abstract

**Importance:**

At birth, neonates with congenital heart disease (CHD) have smaller placentas, lower birth weight, and smaller head circumferences compared with healthy neonates. The onset of feto-placental growth disturbances, however, is not well known.

**Objective:**

To compare fetal body volumes, assess differences in the fetal to placental volume ratios (placental growth relative to the fetus), and investigate the association between in utero fetal body and total brain volume in fetuses with and without CHD.

**Design, Setting, and Participants:**

This case-control study enrolled pregnant women with a fetal diagnosis of CHD and those with healthy pregnancies at Children’s National Hospital in Washington, DC, from April 2018 to July 2023. Fetal magnetic resonance imaging was obtained up to 2 time points during pregnancy. The fetal to placental ratio was calculated using 3-dimensional magnetic resonance image fetal body volumes and placental volumes.

**Exposure:**

In utero environment CHD.

**Main Outcomes and Measures:**

The main outcomes were the trajectories of body, brain, and placental volumetric growth in fetuses with CHD and in control fetuses. Generalized linear regression and mixed-effects models were applied to identify associations for fetal body volume and fetal to placental volume ratios between CHD and control groups adjusting for fetal sex and gestational age at the time of the magnetic resonance imaging scan.

**Results:**

The study included 108 fetuses (59 male [54.6%]), of which 55 were in the healthy control group (with 55 scans), and 53 had CHD (with 77 scans). Fetal body volumes in fetuses with CHD were smaller compared with control fetuses (β = −193.60 [SE, 44.42]; *P* < .001) with larger fetal to placental volume ratios (β = 0.23 [SE, 0.10]; *P* = .02). The total brain volume was smaller in fetuses with CHD compared with control fetuses (β = −10.87 [SE, 5.09]; *P* = .04).

**Conclusions and Relevance:**

In this case-control study of fetuses with and without CHD, those with CHD demonstrated impaired fetal body growth with higher fetal to placental volume ratios and smaller total brain volume, suggesting that placental failure was associated with growth disturbances in CHD, as demonstrated by the large fetal to placental volume ratios. Additional studies should assess the onset and progression of placental dysfunction and how the timing of placental failure may contribute to neurodevelopmental disability in survivors of CHD.

## Key Points

**Question:**

What are the in utero growth trajectories of the placenta, fetal body, and brain and the association between placental, fetal body, and brain growth in fetuses with congenital heart disease compared with healthy control fetuses?

**Findings:**

In this case-control study of 108 fetuses, placental, fetal body, and total brain volumes in fetuses with congenital heart disease were smaller, with larger fetal to placental volume ratios (placental growth relative to the fetus) when compared with healthy control fetuses.

**Meaning:**

These findings suggest that placental dysfunction precedes fetal body and brain growth failure, increasing the risk of fetal brain vulnerability.

## Teaser

This case-control study compares in utero growth trajectories of the placenta, fetal body, and brain in fetuses with congenital heart disease compared with healthy control fetuses.

## Introduction

Fetal growth restriction has been linked to postnatal neurodevelopmental disorders, including motor and sensory disabilities, cerebral palsy, and cognitive and learning disorders.^1,2,3,4,5,6,7,8^ The most common cause of fetal growth restriction is placental insufficiency, in which the transfer of oxygen and nutrients is inadequate to support optimal fetal growth and development.^1,4,9,10,11,12^ In response to placental insufficiency, the fetus compensates by slowing its overall body growth and redistributing cardiac output to essential organs, predominantly the brain.^1,13,14,15,16^ This brain-sparing effect serves to protect fetal brain growth against the failing placenta.^1,17^ The placenta can also adapt to the in utero environment based on the availability of maternal nutrients and oxygen to promote fetal development despite hostile conditions.^18^ Although the placenta provides the critical functions to support fetal development and survival, it remains one of the most poorly understood organs,^19^ with most studies emanating from postnatal assessments.^19,20,21,22,23,24,25^ Moreover, despite the longstanding knowledge that neonates with congenital heart disease (CHD) have abnormal birth anthropometrics compared with healthy neonates,^26,27,28^ even less is known regarding placental development and fetal growth in the setting of CHD.

Longitudinal neurodevelopmental surveillance of survivors of CHD has identified a spectrum of neurodevelopmental disabilities.^29,30^ Early evidence targeted neonatal cardiac surgery as the culprit of brain injury leading to adverse neurodevelopmental outcomes. However, research has shifted the focus to the in utero environment.^27,31,32,33,34,35,36,37^ It is now well established that fetuses with CHD have structurally abnormal brains when compared with age-matched healthy control fetuses.^27,36,37,38^ Furthermore, despite full-term birth, neonates with CHD have structurally immature brains at birth, more similar to a 35-week gestational age premature neonate.^27^ Postnatally, placentas in pregnancies with CHD are small, with high rates of vascular abnormalities.^19,39,40^ However, there is a paucity of studies focusing on in utero fetal body and placental growth and its temporal and mechanistic association with fetal brain growth in CHD.

Using in vivo magnetic resonance imaging (MRI), the primary objectives of this study were to measure the trajectories of in utero volumetric growth of the placenta and fetal body. Secondary objectives were to measure the fetal brain in utero and to explore feto-placental growth relative to physiologic measures of CHD.

## Methods

This case-control study was reviewed and approved by the institutional review board of Children’s National Hospital in Washington, DC. Written informed consent was obtained from all participants. The study followed the Strengthening the Reporting of Observational Studies in Epidemiology (STROBE) reporting guideline.

### Patient Population

Pregnant women with singleton pregnancies of 16 to 38 weeks’ gestation were enrolled in this longitudinal, prospective observational case-control study from April 2018 to July 2023, in which MRI was performed up to 2 time points in the fetal period. Pregnant women in the control group were healthy volunteers with no significant past medical history, including pregnancy-related or chronic conditions, with normal prenatal screening (laboratory and anatomic imaging studies) and no adverse pregnancy outcomes. Control participants were extracted from an institutional review board–preapproved control database.

### Study Design

This is a retrospective study of prospectively collected data. The research protocol included up to 2 serial fetal MRI studies in fetuses with CHD (the first in the second trimester and the second in the third trimester). Both studies were included in the analysis. Women pregnant with fetuses with confirmed CHD were recruited from the Prenatal Cardiology Program at Children’s National Hospital. Exclusion criteria for fetuses with CHD and healthy fetuses were multiple gestation pregnancy, extracardiac anomalies, known or suspected congenital infection or documented chromosomal abnormalities that could independently influence placental–fetal–brain growth, and maternal contraindication to MRI. Enrolled pregnant women whose fetuses were found to have in utero fetal demise, structural brain abnormalities on fetal brain magnetic resonance images, or postnatal confirmation of a genetic syndrome were subsequently excluded from the analysis.

### Fetal Echocardiography

All fetuses with CHD underwent fetal echocardiographic assessment with 2-dimensional (2D) color and pulsed Doppler evaluation as part of the study protocol, including measures of middle cerebral artery pulsatility index (PI), umbilical artery PI, and the cerebroplacental ratio. Echocardiographic studies were completed within 24 hours of MRI for most fetuses and were reviewed by a single attending cardiologist (M.T.D.). Fetal CHD diagnoses were categorized based on heart lesion and primary CHD class to capture common physiologic features (class I, 2-ventricle CHD without aortic obstruction; class II, 2-ventricle CHD with aortic arch obstruction; class III, single ventricle CHD without aortic obstruction; and class IV, single ventricle CHD with aortic obstruction).^41^ For statistical analyses, dextro-transposition of the great arteries was assigned class 0 and analyzed separately, given it is hemodynamically dissimilar to other class I defects.^27^ Three cardiac lesions were of interest given prior publication on birth anthropometrics: dextro-transposition of the great arteries, tetralogy of Fallot, and hypoplastic left heart syndrome.^26^

### Fetal MRI

All MRI scans for fetuses with CHD and control fetuses were performed on a GE Discovery MR450 1.5T scanner (GE Healthcare) using an 8-channel surface receive coil (USA Instruments). Images were acquired in the supine or lateral position based on maternal comfort. Single-shot fast spin echo T2-weighted placental images were performed as follows: fat suppressed with echo time (TE), 160 ms; repetition time (TR), 1100 ms; field of view (FOV), 420 × 420 mm; 4 mm slice thickness; and 40 to 60 consecutive slices for full placental coverage in the axial plane. Placental acquisition time was 45 seconds. 3D Steady-state free precession images of the intrauterine cavity for whole fetal body volume were acquired with the following parameters: TR, 2.8 ms; TE, 1.1 ms; slice thickness, 3 mm; flip angle, 35°; matrix size, 256 × 192; and FOV, 360 × 288 mm. The total sequence acquisition time was between 12 and 14 seconds, based on the size of the fetal body in the sagittal plane. Fetal brain images were acquired with the following parameters: TR, 1100 ms; TE, 160 ms; flip angle, 90°; FOV, 320 × 320 mm; matrix size, 256 × 192; in-plane resolution, 1.25 × 1.66 mm^2^; and slice thickness, 2 mm, with acquisition time of 2 or 3 minutes per plane. Using a previously validated method,^42^ images of axial, coronal, and sagittal planes were reconstructed into high-quality 3D volumetric images of resolution (0.86 × 0.86 × 0.86 mm^3^). No contrast or sedation was used for any of the imaging studies. The same imaging protocol, which has been previously published,^36,43,44,45^ was used for fetuses with CHD and control fetuses.

### Postprocessing Image Segmentation and Volume Calculations

Both groups were blinded and anonymized to avoid potential sources of bias, and all postprocessing was identical between cohorts. The placenta was manually outlined using ITK-SNAP, version 3.8.0 (Penn Image Computing and Science Laboratory),^46^ and the volume was converted to cubic centimeters.^43^ Segmentations of the fetal body were obtained from the described in-house pipeline^44^ and manually corrected as previously described and reported in cubic centimeters^44^ (eFigure 1 in Supplement 1). Fetal total brain volumes (TBVs) were automatically extracted using a previously validated deep learning–based pipeline^36,45^ and reported in cubic centimeters and manually corrected as needed. Placental and whole fetal body segmentations were manually performed by trained scientists (N.A., K.K., and M.J.), and brain corrections were performed by a single scientist (M.J.). Interrater and intrarater reliability were calculated on 20% of the cohort. Placenta interrater reliability was 0.9684, intrarater reliability was 0.9151, fetal body interrater reliability was 0.9850, intrarater reliability was 0.9957, and TBV intrarater reliability was 0.9982. Fetal body to placental volume ratios were calculated by dividing the fetal body volume by the placental volume for both fetuses with CHD and control fetuses.

### Demographic and Clinical Data

Clinical and demographic data were collected through medical chart review and/or maternal questionnaires. Gestational age was calculated based on maternal last menstrual period or the fetuses’ first-trimester ultrasound measurement. Birth weight was corrected for gestational age at birth using the Fenton growth chart calculations.^47^

### Statistical Analysis

Baseline clinical and demographic characteristics are presented. The *t* test analysis for normally distributed continuous variables was performed to assess significant differences in the mean (SD) between fetuses in the control and CHD groups. The Wilcoxon rank sum test was applied to compare non–normally distributed continuous data, a χ^2^ test was used to evaluate categorical variables for statistical significance, and the Fisher exact test was used with contingency table cell counts below 5. Scatterplots were displayed to assess the association between placental volume, fetal body volume, fetal body to placental volume ratios, and TBV with gestational age in CHD and control groups. Generalized linear regression and mixed-effects models were applied to identify associations for fetal body volume and fetal body to placental volume ratios with the CHD and control groups, adjusting for fetal sex and gestational age at the time of the MRI scan. Collinearity checks were performed using variance inflation factors to identify clinical and demographic factors associated with birth weight and head circumference. All *P* values were 2-tailed, with threshold *P* values <.05 considered statistically significant and CIs set at a 95% confidence level. All analyses were performed using RStudio, version 2023.12.1.402 in R (R Project for Statistical Computing).

## Results

### Characteristics of CHD and Control Cohorts

A total of 117 pregnant women were enrolled, among whom 58 had a healthy fetus (control cohort), and 59 had a diagnosis of fetal CHD. Of these, 6 of the fetuses with CHD were excluded due to movement artifacts, rendering pipeline completion and manual segmentation impossible. In the control cohort, 3 fetuses were excluded: 1 due to postnatal genetic diagnosis and 2 due to movement artifacts. The final cohort included 108 fetuses (49 female [45.4%] and 59 male [54.6%]), of which 55 were in the control group, and 53 were in the CHD group. Among the fetuses with CHD, 24 had 2 fetal body MRIs, for a total of 132 scans (55 in the control group and 77 in the CHD group) (eFigure 2 in Supplement 1).

Data for all maternal and fetal demographics are presented in Table 1. Clinical characteristics of the CHD cohort are presented in Table 2. Among the 53 fetuses in the CHD cohort, 33 (62%) were male. Of the pregnant women, 38 (72%) had prenatal genetic testing, whereas prenatal genetic testing was declined by 9 (17%), and 6 of the test results (11%) were unknown or unavailable for the remaining pregnant women. There were no significant differences in maternal educational level or maternal age between the control and CHD cohorts.

**Table 1.** Demographic Characteristics of the Study Cohorts

Characteristic	Study cohort (N = 108)		*P* value
	Control (n = 55)	CHD (n = 53)	
Sex of fetus, No. (%)			
Female	29 (53)	20 (38)	.11
Male	26 (47)	33 (62)	
Gestational age at scan, mean (SD)^a^^,^^b^	28.11 (6.33)	32.16 (3.63)	<.001
Gestational age at birth, mean (SD)^a^	39.60 (1.34)	38.54 (1.22)	<.001
Birth weight, mean (SD), g^a^	3388.00 (512.25)	3036.63 (435.08)	<.001
Birth head circumference, mean (SD), cm^a^^,^^c^	34.60 (1.50)	33.22 (1.53)	<.001
Birth length, mean (SD), in^a^	NA	49.38 (2.30)	NA
Maternal age at scan, mean (SD), y	34.59 (5.74)	32.61 (5.84)	.08
Maternal educational level, No. (%)^d^			
<7th Grade, junior high school, some high school, or high school or trade school graduate	5 (10)	7 (14)	.54
Some college or college graduate	23 (45)	21 (43)	
≥Graduate school	23 (45)	20 (41)	
Maternal medical history, No. (%)			
Diabetes	0	2 (4)	NA
Hypothyroidism	0	2 (4)	
Hyperthyroidism	0	0	
Epilepsy	0	0	
Pregnancy-induced hypertension	0	1 (2)	
Prenatal exposures, No. (%)			
Alcohol	0	0	NA
Cigarettes	0	1 (2)	
Marijuana	0	1 (2)	
Unknown	0	6 (11)	

Abbreviations: CHD, congenital heart disease; NA, not applicable.

^a^ Unadjusted mean (SD).

^b^ Data were reported per scan.

^c^ Data were available for 83 of 108 fetuses (40 in the control group; 43 in the CHD group).

^d^ Data were available for 100 of 108 pregnant women (51 in the control group; 49 in the CHD group).

**Table 2.** Clinical Characteristics of the CHD Cohort

Characteristic	CHD cohort (n = 53)
Primary CHD class, No. (%)	
Class 0 (TGA)	13 (25)
Class I (without TGA)	14 (26)
Class II	9 (17)
Class III	8 (15)
Class IV	9 (17)
CHD lesion, No (%)	
d-TGA (with or without IVS or VSD)	14 (26)
HLHS	9 (17)
TOF (with or without PA or MAPCAs)	7 (13)
HRH	6 (11)
Other functional single ventricle anatomy	2 (4)
HRH, TA, and DORV	1 (2)
HRH and PA	2 (4)
DORV, HRH, and PS	1 (2)
VSD with coarctation	2 (4)
Truncus arteriosus	3 (6)
Truncus with IAA	1 (2)
Ebstein’s anomaly with PA	1 (2)
CAVC, heterotaxy	2 (4)
DORV and coarctation	2 (4)
DORV and PS	1 (2)
AA and VSD	1 (2)
CAVC and coarctation	1 (2)
CAVC	1 (2)
Heterotaxy with complex CHD	2 (4)
Prenatal genetic testing, No. (%)	
Yes	38 (72)
No	9 (17)
Unknown	6 (11)
Estimated fetal weight, mean (SD), g^a^	1985.05 (652.67)
Estimated fetal weight percentile, mean (SD)^a^	66.00 (31.00)
Middle cerebral artery PI, mean (SD)	1.85 (0.42)
Umbilical artery PI, mean (SD)	1.15 (0.28)
Cerebro-placental ratio, mean (SD)	1.85 (1.53)

Abbreviations: AA, aortic arch; CAVC, complete atrioventricular canal; CHD, congenital heart disease; d-TGA, dextro-transposition of the great arteries; DORV, double outlet right ventricle; HLHS, hypoplastic left heart syndrome; HRH, hypoplastic right heart anatomy; IAA, interrupted AA; IVS, intact ventricular septum; MAPCAs, major aorta-pulmonary collateral arteries; PA, pulmonary atresia; PI, pulsatility index; PS, pulmonary stenosis; TA, tricuspid atresia; TOF, tetralogy of Fallot; VSD, ventricular septal defect.

^a^ Based on fetal echo.

### Placental and Fetal Body Volumes and TBVs Across Gestation in Fetuses With CHD Compared With Control Fetuses

The linear mixed-effects model correcting for gestational age at the time of the MRI and fetal sex revealed that placental volumes in the CHD cohort were significantly smaller than in the control cohort across gestation (β = −109.29 [SE, 28.32]; *P* < .001) (Table 3 and Figure, A). Fetal body volumes increased with gestational age for both the control and CHD cohorts (Figure, B). Fetal body volumes with CHD were significantly smaller than control fetal body volumes after correcting for gestational age at the time of the MRI and fetal sex (β = −193.60 [SE, 44.42]; *P* < .001) (Table 3 and Figure, B). Using global analysis of variance testing, the subanalysis revealed no significant difference in fetal body volumes when stratified by CHD class or CHD lesion type (Table 4). Consistent with prior reports, TBV was significantly smaller in the CHD cohort compared with the healthy cohort (β = −10.87 [SE, 5.09]; *P* = .04) (Table 3 and Figure, C).

**Table 3.** Adjusted Association of Total Brain Volume, Placental Volume, Fetal Body Volume, and Fetal Body to Placental Volume Ratio With CHD vs Control^a^

Factors	Total brain volume		Placental volume		Fetal body volume		Fetal body to placental volume ratio	
	β Estimate (SE)	*P* value	β Estimate (SE)	*P* value	β Estimate (SE)	*P* value	β Estimate (SE)	*P* value
CHD vs control	−10.87 (5.09)	.04	−109.29 (28.32)	<.001	−193.60 (44.42)	<.001	0.23 (0.10)	.02
Gestational age at scan	15.74 (0.33)	<.001	38.19 (1.96)	<.001	147.93 (3.42)	<.001	0.12 (0.01)	<.001
Sex (female vs male)	14.90 (4.93)	.003	7.61 (27.38)	.78	56.89 (42.33)	.18	0.15 (0.09)	.12

Abbreviation: CHD, congenital heart disease.

^a^ Linear mixed-effects model adjusting for gestational age at scan and fetal sex.

**Figure.**
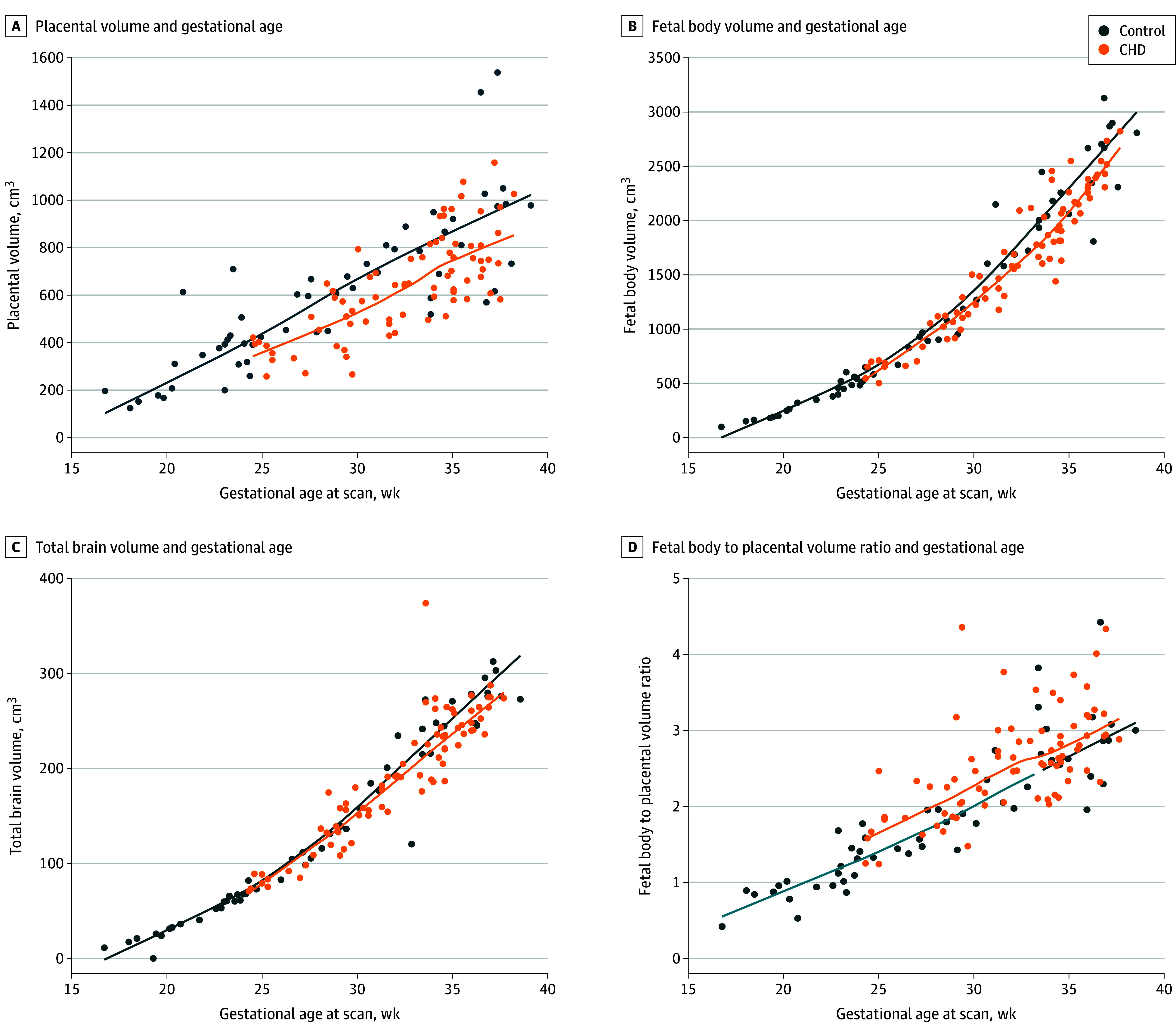
Scatterplots of the Association of Unadjusted (Raw) Placental Volumes, Fetal Body Volumes, Total Brain Volumes, and Fetal Body to Placental Volume Ratios With Congenital Heart Disease (CHD) vs Control Groups Across Gestational Age Solid curves indicate placental volumes, fetal body volumes, total brain volumes, and fetal body to placental volume ratios across gestation for the control group (blue) and the CHD group (orange).

**Table 4.** Association of Fetal Body Volume and Fetal Body to Placental Volume Ratio With CHD Characteristics

Characteristic	Fetal body volume (n = 77)		Fetal body to placental volume ratio (n = 77)	
	Volume	*P* value	Volume ratio	*P* value
CHD class, mean (SD)^a^				
0 (n = 19)	1637.61 (660.39)	.86	2.78 (0.72)	.12
1 (n = 36)	1587.33 (606.87)		2.45 (0.52)	
2 (n = 14)	1768.62 (500.28)		2.86 (0.70)	
3 (n = 12)	1660.67 (688.17)		2.45 (0.51)	
4 (n = 18)	1513.75 (614.88)		2.33 (0.74)	
CHD lesion, mean (SD)^a^				
d-TGA (n = 19)	1641.21 (641.98)	.85	2.83 (0.73)	.05
TOF (n = 10)	1499.30 (525.30)		2.39 (0.48)	
HLHS (n = 27)	1592.52 (645.69)		2.35 (0.64)	
Echocardiographic measure, difference^b^				
MCA PI	−0.17	.21	−0.16	.24
UA PI	−0.11	.41	−0.08	.54
CPR	−0.01	.94	−0.11	.45

Abbreviations: CHD, congenital heart disease; CPR, cerebro-placental ratio; d-TGA, dextro-transposition of the great arteries; HLHS, hypoplastic left heart syndrome; MCA, middle cerebral artery; PI, pulsatility index; TOF, tetralogy of Fallot; UA, umbilical artery.

^a^ Global analysis of variance testing was used.

^b^ Pearson correlation was used.

### Relative Growth of Placenta, Fetal Body, and TBVs in Fetuses With CHD Compared With Control Fetuses

Fetal to placental volume ratios were significantly larger at all gestational ages in the CHD cohort than in the healthy cohort (β = 0.23 [SE, 0.10]; *P* = .02) (Table 3 and Figure, D). Global analysis of variance testing revealed no significant difference in fetal to placental volume ratios when stratified by CHD class or cardiac lesion type (Table 4). There was no association between middle cerebral artery PI, umbilical artery PI, and cerebroplacental ratio with fetal body or fetal to placental volume ratios (Table 4). There was no significant difference in the association between fetal body volume and TBV with the CHD and control groups, with fetal body and fetal brain growth increasing at consistent rates throughout gestation in both cohorts.

## Discussion

In this case-control study, we demonstrate that placental, fetal body, and TBV in fetuses with CHD were significantly smaller than were those in healthy control fetuses. Despite smaller body volumes, fetal to placental volume ratios were larger in pregnancies with CHD compared with healthy control pregnancies, highlighting an imbalance between fetal and placental growth. Consistent with prior studies, TBV was smaller in the CHD cohort.^34,36,38^ Although underpowered, subanalysis of fetal to placental volume ratios and CHD lesion type suggests that specific cardiac lesions may be associated with placental dysfunction.

CHD is known to be associated with disturbed growth, including small for gestational age at birth and low birth weight^17,48^; 1 study identified a 3-fold increased risk of fetal growth restriction with a prenatal diagnosis of CHD.^49^ However, growth in CHD has often been defined using anthropometric measures collected at birth as a surrogate of fetal growth in fetuses with CHD regardless of placental pathology.^32,49^ We demonstrate significantly smaller intrauterine fetal body volumes across gestation when compared with healthy controls. Despite neonatal anthropometric growth differing based on CHD lesion type,^26^ in this study, there were no significant differences when stratified by CHD class or cardiac lesion type. The purpose of the subgroup analysis, although underpowered, was to explore the associations of fetal hemodynamics, placental function, and fetal growth; as a hypothesis-generating objective, these results may better inform future study design.

Animal studies have demonstrated that the placenta adapts its nutrient transfer efficiency by functional and morphologic modifications for optimal fetal growth.^50^ Failure of placental adaptations results in a fetus not meeting its genetic growth potential.^50^ The birthweight to placental weight ratio is often used to define placental efficiency, representing the grams of fetus produced per gram of placenta.^50^ Mirroring this concept, we used fetal to placental volume ratios as an in vivo surrogate to evaluate the growth of the fetus and placenta relative to one another. In this cohort, placental volumes were smaller in CHD, with larger fetal to placental volume ratios when compared with healthy pregnancies. The large ratios in the CHD cohort represent a growth imbalance, and, although underpowered, subanalysis between fetal to placental volume ratios and CHD lesion type suggests that fetuses with specific cardiac lesions may be particularly vulnerable to placental dysfunction. This finding should be further explored in larger cohorts to better assess the significance of these findings. Matsuda et al^51^ explored the concept of balanced and unbalanced pregnancies using normalized birthweight to placental weight ratios and revealed that unbalanced pregnancies with disproportionate fetal and placental growth were associated with worse perinatal outcomes. Indeed, one of the largest studies, to our knowledge, on birthweight to placental weight ratios^52^ demonstrated elevated odds ratios for fetal death in both the lowest and highest birthweight to placental weight ratio quartiles.^50^ Conversely, fetal growth restriction secondary to placental insufficiency typically presents with smaller birthweight to placental weight ratios, which suggests that placental dysfunction and disrupted feto-placental growth in CHD do not mirror typical patterns of placental insufficiency despite the associated risk of adverse perinatal outcomes.^52^

Visual inspection of the data suggests that both fetal body and TBV growth in fetuses with CHD may diverge from healthy control fetuses at a similar gestational time point. This represents a time of exponential fetal growth, particularly for the developing brain.^34,53,54^ This normal acceleration in fetal brain growth is supported by increased cerebral oxygen and nutrient delivery in the form of increased blood flow, which is dependent on appropriate placental development. Placental dysfunction is thought to develop gradually, allowing the fetus to adapt to inadequate transplacental nutrients by reducing its growth rate.^55^ A divergence in growth trajectories in the early third trimester may reflect an inability of the placenta to support the increased fetal growth demands, but these observations require further investigations in a larger cohort.

Previous studies into the origins of fetal CHD development have often overlooked the synergistic link between heart and placental development.^56^ The placenta and heart develop concurrently in the first trimester of pregnancy and share common developmental pathways; defective early morphogenesis of these organs is increasingly considered a link between CHD and placental failure.^56^ In our study, the data did not detect an association between in utero growth and specific CHD lesion type; although this may suggest that placental failure is universal in CHD regardless of lesion type, more specific associations may not have been detected due to the small size of the subgroups. We speculate that early developmental disturbances in placental (and cardiac) development may not be exposed functionally until the accelerated third-trimester fetal growth demands. Furthermore, we posit that the in utero circulatory differences based on CHD lesion type^17^ may be associated with restricted fetal body and brain growth.

### Limitations

This study has some limitations. Because CHD is often diagnosed after 20 weeks’ gestation, evaluation of placental volumes, fetal body volumes, and TBVs prior to 20 weeks’ gestation was lacking. Prenatal genetic testing was based on clinically acquired data, and although all fetuses with known genetic syndromes were excluded, those with delayed diagnoses may have been inadvertently included. None of the control fetuses in this cohort had 2 time points of MRI imaging, limiting our ability to compare within-participant growth between the fetuses with CHD and the control fetuses. Exclusion criteria for the control cohort included maternal comorbidities, which was not an exclusion criterion for the CHD cohort. Although relatively few pregnant females in the CHD cohort reported significant medical comorbidities, maternal health may nonetheless be a factor in placental function independent of CHD. Similarly, other reasons for feto-maternal–placental wellness, including social determinants of health, were not fully captured. We were unable to account for many potential confounders; thus, future studies should consider the addition of detailed maternal medical, psychosocial, and environmental comorbidities that were unavailable for this study. The study was underpowered to detect associations between fetal body volumes and fetal to placental volume ratios by CHD class and lesion type given the sample size; this warrants future investigation with a larger cohort. Finally, this study was completed at a single center, so it may lack generalizability.

## Conclusions

In this case-control study, placental volumes, fetal body volumes, and TBVs in fetuses with CHD were smaller, with larger fetal to placental volume ratios throughout gestation, when compared with healthy fetuses. Additional studies are necessary to assess whether placental failure is a significant driver of fetal growth failure in CHD. If confirmed, placental failure may independently increase fetal brain vulnerability and may contribute to the neurodevelopmental disability that is prevalent in survivors of CHD. Nonetheless, the association between placental development and function as it relates to fetal growth is dynamic and complex; ongoing studies with a larger cohort are needed to fully capture the potential drivers of fetal growth, as well as the onset and progression of placental dysfunction.

## Notes

Supplement 1. **eFigure 1.** MRI Segmentations of Fetal Body (Red), Fetal Brain (Blue), and Placenta (Magenta) at Approximately 25 Weeks (A-C) and at Approximately 34 Weeks (D-F) **eFigure 2.** Cohort

Supplement 2.
Data Sharing Statement
