# Supplementary material for: Placental and Fetal In Utero Growth Among Fetuses With Congenital Heart Disease
Source: JAMA Netw Open. 2025 Apr 24;8(4):e257217. doi: 10.1001/jamanetworkopen.2025.7217 (PMC12022807; doi:10.1001/jamanetworkopen.2025.7217)
Supplement: Supplement 1. — eFigure 1. MRI Segmentations of Fetal Body (Red), Fetal Brain (Blue), and Placenta (Magenta) at Approximately 25 Weeks (A-C) and at Approximately 34 Weeks (D-F) eFigure 2. Cohort [file jamanetwopen-e257217-s001.pdf]

Supplemental Online Content

Jacobwitz M, Kapse K, Ngwa J, et al. Placental and fetal in utero growth among fetuses with congenital heart disease. *JAMA Netw Open*. 2025;8(4):e257217. doi:10.1001/jamanetworkopen.2025.7217

eFigure 1. MRI Segmentations of Fetal Body (Red), Fetal Brain (Blue), and Placenta (Magenta) at Approximately 25 Weeks (A-C) and at Approximately 34 Weeks (D-F)
eFigure 2. Cohort

This supplemental material has been provided by the authors to give readers additional information about their work.

© 2025 Jacobwitz M et al. *JAMA Network Open*.

eFigure 1. MRI Segmentations of Fetal Body (Red), Fetal Brain (Blue), and Placenta (Magenta) at Approximately 25 Weeks (A-C) and at Approximately 34 Weeks (D-F)

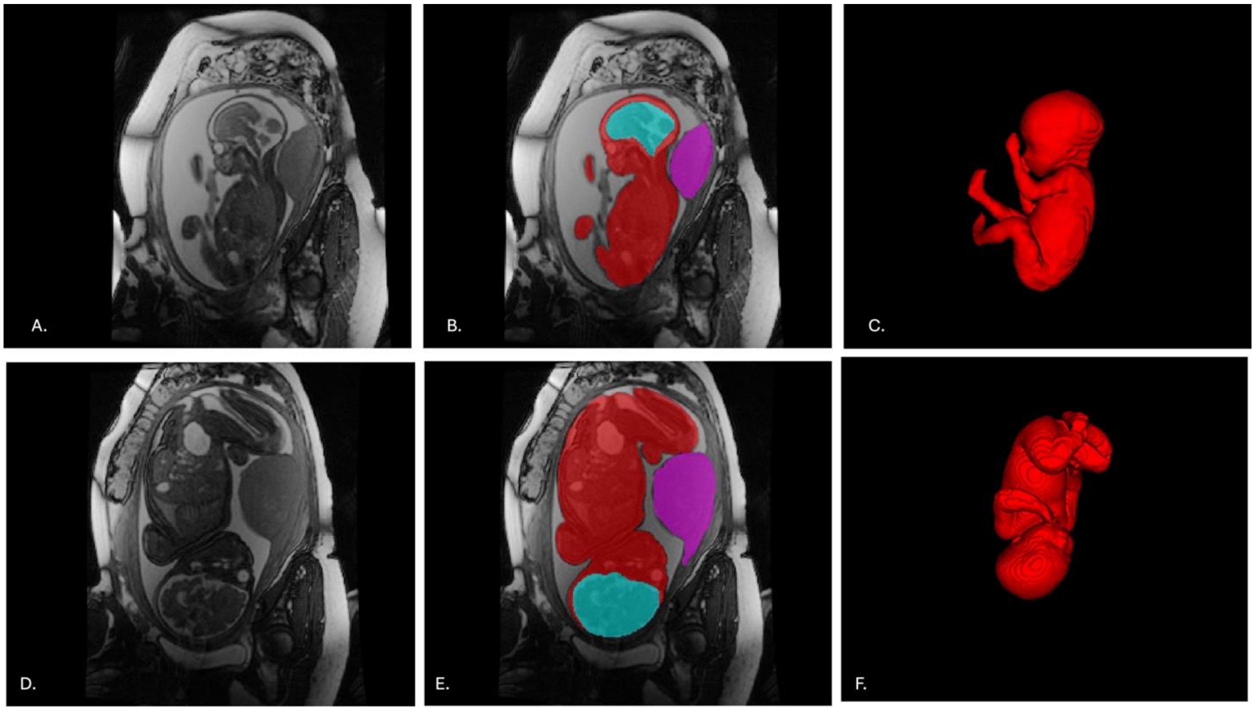

© 2025 Jacobwitz M et al. *JAMA Network Open*.

eFigure 2. Cohort

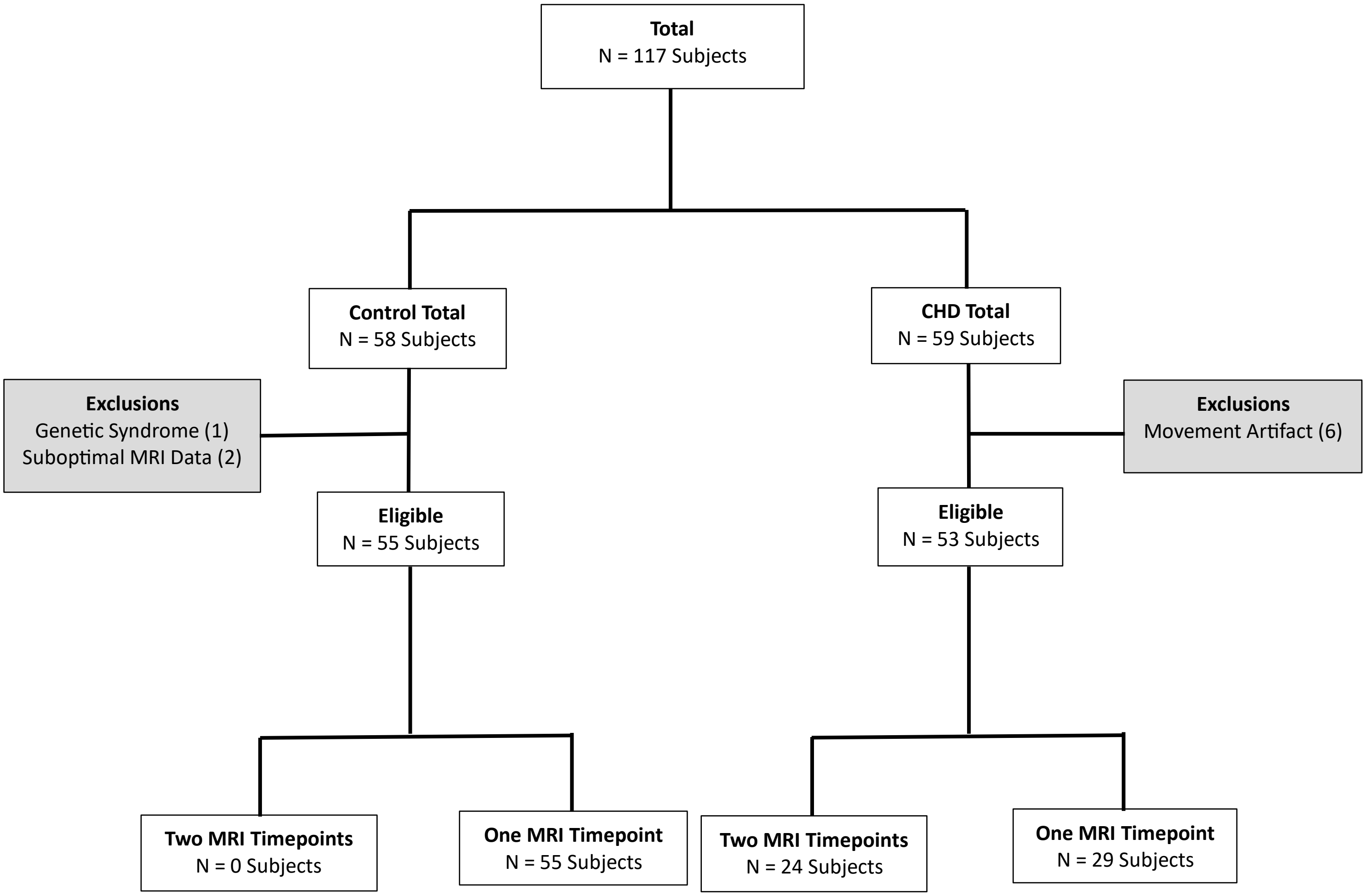

© 2025 Jacobwitz M et al. *JAMA Network Open*.
