# Supplementary material for: Placental and Fetal In Utero Growth Among Fetuses With Congenital Heart Disease
Source: JAMA Netw Open. 2025 Apr 24;8(4):e257217. doi: 10.1001/jamanetworkopen.2025.7217 (PMC12022807; doi:10.1001/jamanetworkopen.2025.7217)
Supplement: Supplement 2. — Data Sharing Statement [file jamanetwopen-e257217-s002.pdf]

Data Sharing Statement

Data
Data available: No

Additional Information
Explanation for why data not available: Data is available upon request to corresponding author.
